# Supplementary material for: Neuronal activity patterns in microcircuits of the cerebellar cortical C3 zone during reaching
Source: J Physiol. 2022 Nov 9;600(23):5077–99. doi: 10.1113/JP282928 (PMC10099968; doi:10.1113/JP282928)
Supplement: Supplementary file 1 — Statistical Summary Document [file TJP-600-5077-s002.docx]

**Manuscript Title:** Neuronal activity patterns in microcircuits of the cerebellar cortical C3 zone during reaching

**Authors:** Nadia L Cerminara, Martin Garwicz, Henry Darch, Conor Houghton, Dilwyn E Marple-Horvat, Richard Apps

**Animal model used, if applicable:** Cat.

**Underlying hypothesis:** This investigation tests the hypothesis that whether there are systematic differences in patterns of activity between neurons located in different individual microzones of the cerebellum relate to different aspects of a voluntary movement, or whether all microzones within a given zone display the same general pattern of activity.

**Definitions of ‘n’:**

Question 1 n = individual units from multiple animals

Question 2 n = individual units from multiple animals

Question 3: n = individual units from multiple animals.

Question 4: n = individual units from multiple animals.

Question 5: n = individual units from multiple animals

Question 6: n = individual units from multiple animals

Question 7: n = number of microzone classes

Question 8: n = individual units from multiple animals

Question 9: n = individual units from multiple animals

**Statistical summary table:**

| Experimental question number* | Finding/ conclusion | Experimental location/ variable  e.g. muscle, neocortex or genotype | Mean value  (or other summary statistic) | SD | n val. | P** | Units | Data comparisons  e.g. WT vs KO | Statistical test | Any other variable  e.g. subjects’ age or sex | Figure/ table in which data are presented | Comments  e.g. observation |
| --- | --- | --- | --- | --- | --- | --- | --- | --- | --- | --- | --- | --- |
| 1 Forelimb receptive field classification | Could identify 9/10 previously identified C3 microzones | C3 zone of the paravermal cerebellum | - | - | Class 1 = 12  Class 2= 6  Class 3= 6  Class 4= 4  Class 5=2  Class 6=9  Class 7 =0  Class 8=10  Class 9= 17  Class 10 = 7 | - | unit | X | observation | - | 3 | Observation Receptive fields, not quantified. |
| 2 Comparison of size of receptive fields of simple spikes and complex spikes from same Purkinje cell with similar and differing receptive fields | Area of overlap between similar SS and CS peripheral receptive fields are similar | C3 zone of the paravermal cerebellum | 64.4 | 35.6 | 6 | - | % | X | observation |  | 4 | Observation not quantified |
|  |  |  | 2.2 | 3.8 | 3 | - | % | X | observation |  |  |  |
| 3. Comparison of baseline firing rates between classes for which neural data was available | Baseline firing rate between classes was similar | C3 zone of the paravermal cerebellum | class 1 = 32.4  class 2 22.6  class 6 37.6  class 8 31.6  class 9 33.9  class 10 36.7 | class 1= 15.5  class 2 = 13.4 Hz  class 6= 7.2  class 8 =16.5  class 9= 30.1  class 10 = 21 | class 1 = 8  class 2 =4  class 6 = 5  class 8 =7  class 9 =10  class 10 =4 | 0.954 | Hz | Class 1 v class 2  Class 1 v class 6  Class 1 v class 8  Class 1 v class 9  Class 1 v class 10  Class 2 v class 6  Class 2 v Class 8  Class 2 v class 9  Class 2 v class 10  Class 6 v class 8  Class 6 v Class 9  Class 6 v Class 10  Class 8 v class 9  Class 8 v class 10  Class 9 v class 10 | One way ANOVA | - | 6 |  |
| 4. Comparison of changes in firing rates | Both units that increased and decreased their firings rates differed from baseline | C3 zone of the paravermal cerebellum | Increase: baseline = 35.2  peak = 60  decrease: baseline = 23.8 10.9,  peak =11.7 | Increase baseline =24.5; peak =42.1  decrease: baseline = 10.9,  peak =7.8, | Increase =30  Decrease =8 | p<0.0001 | Hz | Increase baseline v peak  Decrease baseline v peak | paired t test | - | 6 |  |
| 5. Comparison of onset and peak latencies between classes of microzones | Differences between onset latency for classes 1 and 6 compared with class 10 | C3 zone of the paravermal cerebellum | ONSET  class 1= -110  class 2 = 0  class 6= -152  class 8= 41  class 9 = -8  class 10= 180  PEAK  class 1= -57.5  class 2 = 60  class 6= --24  class 8= 151  class 9 = -41  class 10= 195 | ONSET  class 1=63  class 2 = 165  class 6=83  class 8= 159  class 9 =180  class 10= 164  PEAK  class 1= 171  class 2 = 148  class 6= -64  class 8= 90  class 9 = 152  class 10= 181 | ONSET AND PEAK  class 1=8  class 2 = 4  class 6=5  class 8= 7  class 9 =10  class 10= 4 | ONSET  p = 0.015  PEAK  p=0.122 | ms | Class 1 v class 2  Class 1 v class 6  Class 1 v class 8  Class 1 v class 9  Class 1 v class 10  Class 2 v class 6  Class 2 v Class 8  Class 2 v class 9  Class 2 v class 10  Class 6 v class 8  Class 6 v Class 9  Class 6 v Class 10  Class 8 v class 9  Class 8 v class 10  Class 9 v class 10 | one-way ANOVA  post hoc Tukey’s |  | 6 | ONSET  class 1 vs class 10 p=0.025, class 6 vs class 10 p=0.017 |
| 6. Reach Duration between microzones | No difference in reach duration was found between classes | C3 zone of the paravermal cerebellum | class 1= 346  class 2= 326  class 6 295  class 8= 271  class 9= 318  class 10= 251 | class 1 =-132ms  class 2= 126  class 6= 115  class 8= 115  class 9= 87  class 10= 82 | 6 | p=0.718  p=0.882 | class | Class 1 v class 9  Class 1 v class 10  Class 2 v class 6  Class 2 v Class 8  Class 2 v class 9  Class 2 v class 10  Class 6 v class 8  Class 6 v Class 9  Class 6 v Class 10  Class 8 v class 9  Class 8 v class 10  Class 9 v class 10 | one-way ANOVA  ANCOVA | For ANCOVA class, average firing rate and reach duration | - |  |
| 7. Baseline and Peak firing rates of fast and slow reaches | no difference between fast and slow reaches | C3 zone of the paravermal cerebellum | baseline fast= 35.1  baseline slow= 37.1  peak fast = 92.3  peak slow = 81.5 | baseline fast= 21  baseline slow= 20.2  peak fast = 56.1  peak slow = 51.4 | 6 | baselinep=0.867 peak p=0.738 | Hz | Baseline fast vs slow  Peak fast vs slow | unpaired t-test |  | 7 |  |
| 8. Time to peak for fast and slow reaches | no difference was found for the latency between fast and slow trials | - | - | - | 6 | r=-0.15, p=0.779 | ms | Fast vs slow | Pearson correlation |  | 7 |  |
| 9. Onset and peak latencies for peak and distal microzoness | difference in onset and peak latencies | C3 zone of the paravermal cerebellum | proximal onset = 135.6  distal onset = -57 proximal peak = 193.  distal peak = 27.5 | proximal onset = 15.8  distal onset = 160.6 ms  proximal peak =121  distal peak = 136.6 | Proximal = 9  Distal = 20 | Proximal p = 0.005  Peak  p=0.01 | ms | Onset proximal vs distal  Peak proximal vs distal | Mann-Whitney |  | 9 | Difference between onset and peak latencies |

*You may use multiple lines for the same question to indicate multiple comparisons

** Authors may wish to make the text bold where p is considered significant against a stated confidence limit.
