# Supplementary figures and images for: Neuronal activity patterns in microcircuits of the cerebellar cortical C3 zone during reaching
Source: J Physiol. 2022 Nov 9;600(23):5077–99. doi: 10.1113/JP282928 (PMC10099968; doi:10.1113/JP282928)

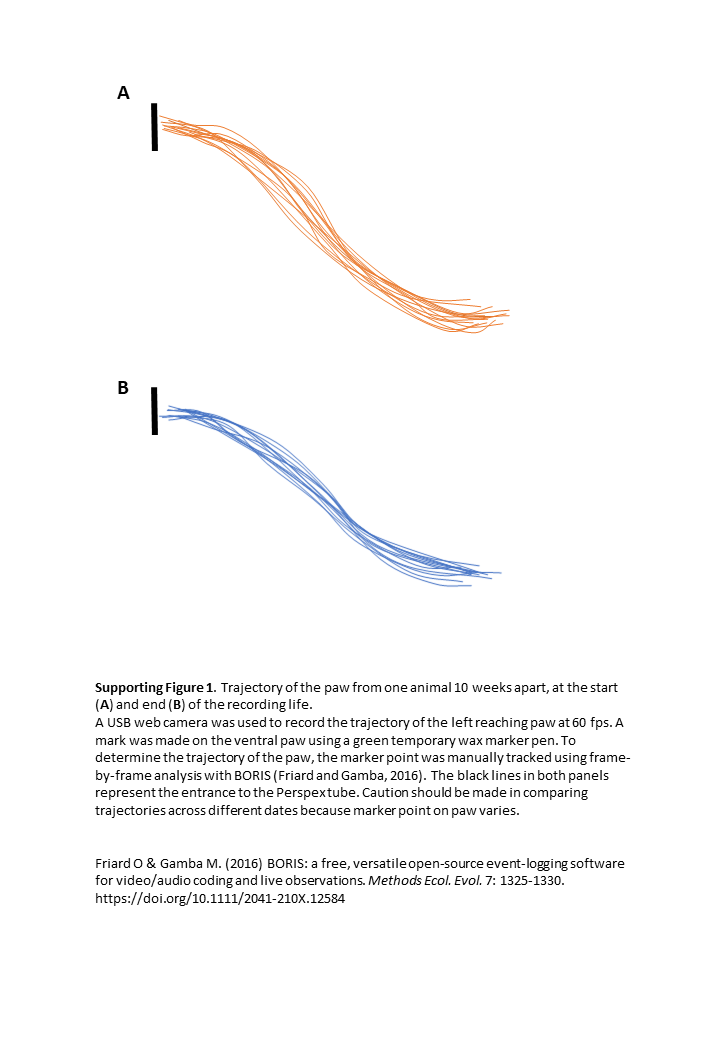

Supplement: Supplementary file 3 — Figure S1 [file TJP-600-5077-s003.tif]
